# Life-Course Relationship between Socioeconomic Circumstances and Timing of First Birth in a Birth Cohort

**DOI:** 10.1371/journal.pone.0170170

**Published:** 2017-01-13

**Authors:** Thea van Roode, Katrina Sharples, Nigel Dickson, Charlotte Paul

**Affiliations:** Department of Preventive and Social Medicine, University of Otago Medical School, Dunedin, New Zealand; TNO, NETHERLANDS

## Abstract

**Objectives:**

This study examines the influence of socioeconomic circumstances in childhood (childhood SES) and adulthood (adult SES) on timing of first birth by age 37.

**Methods:**

A longitudinal study of a 1972–1973 New Zealand birth cohort collected information on socioeconomic characteristics from age 3–32 and reproductive histories at 21, 26, 32 and 38; information on first birth was available from 978 of the original 1037. Relative Risks (RR) and 95% Confidence Intervals (CI) were calculated using Poisson regression to examine first live birth prior to age 21, from 21–25, from 26–31, and from 32–37, by socioeconomic characteristics at different ages.

**Results:**

Overall, 68.5% of men had fathered a child and 75.9% of women had given birth, by age 37; with overall differences in parenthood to age 31 for men, and 37 for women evident by childhood SES. While parenthood by age 20 was strongly associated with lower childhood SES for both sexes, first entry into motherhood from 32–37 was more likely with higher adult SES at age 32 (RR = 1.8, 95% CI 1.1–3.0 for medium and RR = 1.9, 95% CI 1.1–3.3 for high compared with low). Education also differientated age at parenthood, with those with higher education more likely to defer fatherhood past age 31, and motherhood past age 25 followed by a period of increased likelihood of motherhood for women with higher levels of education from age 32–37 (RR = 1.4, 95% CI 0.87–2.2 and RR = 1.7, 95% CI 1.1–2.6 for medium and high respectively compared with low).

**Conclusions:**

SES varies across the lifecourse, and SES at the time has the strongest association with first births at that time. Low childhood SES drives adolescent parenthood, with resulting cumulative differences in parenthood past age 30. Those with more education and higher adult SES are deferring parenthood but attempt to catch up in the mid to late thirties.

## Introduction

Postponement of childbearing is a widespread phenomenon, with many of the more developed countries exhibiting mean ages at birth well exceeding previous generations [1–3]. Despite this overall shift towards later childbearing, there is still substantial variation in age at first birth within many countries, that may be influenced by socioeconomic circumstances in both childhood and adulthood [3,4]. A careful understanding of any socioeconomic differentials in age at first birth is important given that early parenthood has been associated with increased disadvantage for children while increasing age is associated with reduced fecundity [5,6].

Surveys and comparisons of aggregate data suggest that variation in age at first birth is more pronounced in countries with ‘liberal’ economies and more limited social policies to support families compared to ‘universalistic’ regimes, with increasing heterogeneity in the former according to socioeconomic circumstances [7–11]. The extent that socioeconomic circumstances truly drive variation in age at first birth is difficult to ascertain, as the use of measures of socioeconomic circumstances from one point in time may result in bias. Causal relationships may be distorted where socioeconomic factors in adulthood are measured at the time of interview, rather than prior to conception, or where data are aggregated at the country level [12–14].

Furthermore, it is unclear whether socioeconomic circumstances influence fatherhood in a similar way, as data for men are limited, and whether socioeconomic circumstances in childhood contribute to these observed patterns. A life-course approach is required that is able to investigate the influence of both childhood and adult socioeconomic circumstances on entry into parenthood for both sexes.

Notably, the term postponement implies eventual entry into parenthood. However, whether postponement is a delay that will be followed by a recuperation phase where individuals go on to become parents, or whether more will remain childless is also less clear [15–17]. Cohort approaches support the idea that there will be some recuperation, however, as many individuals are now delaying parenthood to ages where natural fertility is declining for both men and women, this could result in a greater proportion of the population not being able to become parents or have as many children as they desire [17–21].

The shift to delayed parenthood has occurred within societies that have been characterised as ones where individual fulfilment is paramount, where women’s roles in the labour force have gained increasing importance, and where social norms have become more diverse regarding family structures and sizes and the importance of becoming a parent at all [22–25]. The widespread use of effective contraceptives allows individuals to weigh the personal benefit of having a child amidst competing lifestyle choices [1]. Economic theorists suggest parenthood is also influenced by costs and ‘opportunity costs’ associated with having children that include the direct expenses of raising children, and lost career opportunities or income [26–29]. These costs may vary by gender and be higher for those with higher socioeconomic status, or for those with higher levels of education, who may also seek to establish careers prior to becoming parents, while enrolment in education may be perceived as incompatible with parenthood [1]. While higher levels of education have been associated with increased postponement of childbearing overall, the impact of level of education on timing of birth throughout the life-course needs to be examined [12,13]. Associations between low adult socioeconomic status and earlier parenthood have been demonstrated, but they vary among countries [8].

Socioeconomic circumstances in childhood also influence timing of parenthood, with lower socioeconomic status of the family in childhood associated with adolescent pregnancy and parenthood [11, 30–34]. This relationship in adolescence is presumed to operate through pathways such as ‘intergenerational transmission’ of social norms for earlier childbearing, or less parental control [35]. This association with lower socioeconomic status of the family and earlier parenthood appears to persist into the mid-twenties, with suggestions that ongoing differences may be due to differences in education at later ages [4,33,36–38]. However, the long term impact of childhood socioeconomic status needs to be investigated as there are few representative samples with data for men and women that examine the relationship with parenthood at later ages, or that investigate whether any ongoing differences in adulthood operate primarily through socioeconomic circumstances in childhood or adulthood.

The Dunedin Multidisciplinary Health and Development Study is well positioned to examine these issues as it is a longitudinal study of a birth cohort of men and women, with prospective data on socioeconomic circumstances, and full reproductive histories, collected at several ages to 38 years. Prior analyses of this cohort demonstrated that postponement past age 30 was common, and there was a relationship between early parenthood and low socioeconomic status of the family in childhood [5,37–39]. Here we examine the effects of socioeconomic status in childhood and adulthood, and education, on entry into parenthood up to age 38. Our primary aim is to examine the main effects of socioeconomic status and education on first birth at different ages, using socioeconomic measures from each time period that directly precede the births. Furthermore, we aim to assess the long term impact of socioeconomic status in childhood on age at entry into parenthood up to age 38, and to consider the independent effects of childhood and adult socioeconomic status, and of adult socioeconomic status and education.

## Materials and Methods

### The study

Participants are members of the Dunedin Multidisciplinary Health and Development Study. The longitudinal cohort consists of all individuals born between April, 1972 and March, 1973 in Dunedin, New Zealand, who lived in the province and participated in the first follow-up assessment at age 3 (N = 1,037; 52% male) [40]. Assessments have been carried out at ages 3, 5, 7, 9, 11, 13, 15, 18, 21, 26, 32 and 38. Ethical approval for Phase (age) 38 of the Dunedin Multidisciplinary Health and Development Study was granted by the Lower South Regional Ethics Committee of the Ministry of Health (New Zealand) [LRS/10/03/012]. Written informed consent was obtained from all participants. At age 21, compared to their age group in the country as a whole, many demographic features were similar, but the sample had a slightly higher level of educational achievement and fewer people of Maori ethnicity [41].

### Reproductive histories

Reproductive histories were sought through a computer-presented questionnaire at ages 21, 26, 32 and 38, and were consistent across assessments. Study members were asked about pregnancies ‘ever’ at 21 and 26, and ‘since the previous assessment’ at 32 and 38, the age at the start of each pregnancy, and the outcome. At the age 38 assessment, study members were asked about desire to have children in the future.

Data from multiple assessments were used to determine the occurrence of a conception leading to a first live birth within four age periods: prior to age 21, from 21–25, from 26–31, and from 32–37. These periods were used to ensure that the outcome of all conceptions at each assessment was known.

### Socioeconomic measures

Information on socioeconomic circumstances in childhood (childhood SES) was provided by the parents at the age 3, 5, 7, 9, 11, 13, and 15 assessments. Childhood SES was based on parental occupation to the age 15 assessment, averaged across assessments using the highest level of socioeconomic status of either parent at each assessment, using the Elley-Irving scale [42,43]. This is a six-point scale which assigns parents’ self-reported occupational status into one of six categories on the basis of the educational levels and income associated with that occupation using data from the New Zealand census.

Information on personal socioeconomic status in adulthood based on occupation (adult SES) and level of education was collected from study members at 21, 26, and 32. Adult SES at age 21 and 26 was defined using the aforementioned Elley-Irving. Adult SES at age 32 was defined using the New Zealand Socioeconomic Index (NZSEI) [44]. This replaced the Elley-Irving scale and is similarly constructed using educational level and income to rank occupations.

Level of education was based on highest educational qualification obtained by each assessment, and grouped as ‘low’ (school certificate or less), ‘medium’ (higher school certificate), and ‘high’ (university bursary) at age 21 and as ‘low’ (high-school or less), ‘medium’ (post-secondary, not university), and ‘high’ (university) at ages 26 and 32.

### Analysis

Stata version 12.0 SE was used for all analyses. The distribution of the cohort on socioeconomic status at four ages was compared for men and women, and socioeconomic status across these four ages was plotted to assess movement between levels of socioeconomic status over time [45]. For the first age period (before 21) childhood socioeconomic status of the family was used.

The proportion to report a first birth was given, as well as the distribution by socioeconomic characteristics, for those with no birth by the start of each of the four age periods.

The cumulative incidence of first birth by age 37 was calculated by childhood SES, for each sex. The cumulative incidence of first birth was then calculated for each age period, by each socioeconomic characteristic at the start of the period. Only those who had not had a live birth in the prior age periods were included in the risk set for each age period. Poisson regression was used to estimate the Relative Risks (RR) and 95% Confidence Intervals (95% CI) with robust standard errors to account for the binary outcome. Separate regressions were fitted for each age period. To assess whether the pattern varied by age, all age periods were included in a single Poisson regression, modelled with robust standard errors. Age period was included as a main effect and in interaction terms, with study members clustered on identification number. The Wald test was used to obtain p-values for interaction. Multivariate models were then constructed which included a) both childhood SES and adult SES, and b) adult SES and level of education.

## Results

**Fig 1:**
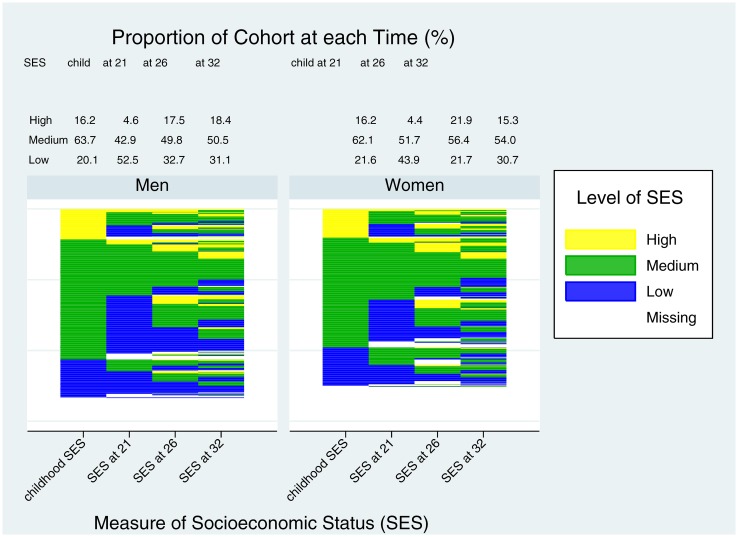
Changes in Socioeconomic Status (SES) over Time: Individual change (Figure) and distribution of cohort (%) by sex. The Figure shows individual change in SES at four ages for men and women separately. Above the figure for each time period, the proportion of men and women in the cohort with low, medium, and high SES is also given.

Of the original cohort of 1037, information on childhood SES was available from 1031, and on adult SES from 977 at the age 21 assessment, 936 at the age 26 assessment, and 971 at the age 32 assessment. Fig 1 displays changes in socioeconomic status for the entire cohort across the life-course. Changes in the pattern represent individual change in socioeconomic status from one period to the next. Notably, the figure shows considerable change from one age period to the next demonstrating the substantial degree of movement for individuals between periods. Comparing whether childhood SES was similar to adult SES at the age 32 assessment showed that only 50.0% of men from families with low status had low adult status at the age 32 assessment, 53.1% from families of medium status had medium status, and even less, 27.3% from families of high status had high status. The respective proportions for women were 50.0%, 57.3%, and 26.9%. Overall, the socioeconomic distributions for the whole cohort were most similar when comparing the measure from childhood and the age 32 assessment.

**Fig 2:**
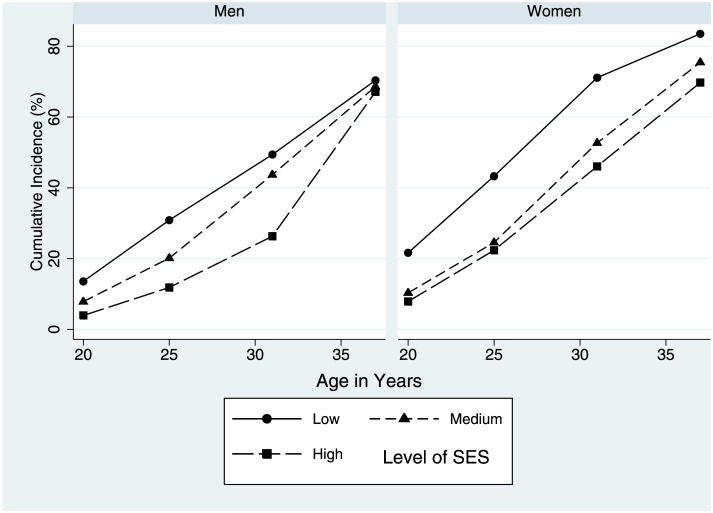
Cumulative Incidence of First Birth by Childhood Socioeconomic Status (SES).

Table 1 gives the number to report a first live birth within each age period, and provides the socioeconomic distribution for the subgroup of the cohort with no first birth at the start of each period. The cumulative incidence of live birth by age 21, 25, 31 and 37 increased from 8.2% by age 20, to 68.5% by age 37 for men and 12.3% to 75.9% for women. Fig 2 displays the cumulative incidence of first live birth by childhood SES. For both men and women, the cumulative incidence of first birth was higher to age 31 for those from families of lower socioeconomic status, with a gradient present. By age 37 this difference by socioeconomic status remained for women but not men. For those with no live birth by the age 38 assessment, 52.9% of men and 46.8% of women reported that they wanted to have a child in the future, 24.6% of men and 22.9% of women were undecided, and 22.5% of men and 30.3% of women did not want to do so (Table 1).

**Table 1:** Distribution of first births and socioeconomic characteristics in the cohort over four age periods.

*Age period*		Men	Women
		n (%)	n (%)
*Prior to 21*	**Total assessed on first birth**	**499 (100.0)**	**479 (100.0)**
	**First birth by age 20**	**37 (7.4)**	**56 (11.7)**
	**Childhood socioeconomic status**^a^	**497 (100.0)**	**476 (100.0)**
	Low	95 (19.1)	103 (21.6)
	Medium	323 (65.0)	294 (61.8)
	High	79 (15.9)	79 (16.6)
*From 21–25*	**Total assessed on first birth with no birth by age 20**^a^	**444 (100.0)**	**417 (100.0)**
	**First birth from age 21–25**	**56 (12.6)**	**72 (17.3)**
	**Level of education at age 21**^a^^b^	**434 (100.0)**	**414 (100.0)**
	Low (School certificate or less)	137 (31.6)	101 (24.4)
	Medium (Higher school certificate)	175 (40.3)	184 (44.4)
	High (University bursary)	122 (28.1)	129 (31.2)
	**Adult socioeconomic status at age 21**^a^	**433 (100.0**	**413 (100.0)**
	Low	219 (50.6)	179 (43.3)
	Medium	192 (44.3)	213 (51.6)
	High	22 (5.1)	21 (5.1)
*From 26–31*	**Total assessed on first birth with no birth by age 25**^a^	**371 (100.0)**	**336 (100.0)**
	**First birth from age 26–31**	**95 (25.6)**	**125 (37.2)**
	**Level of education at age 26**^a^	**371 (100.0)**	**336 (100.0)**
	Low (High school or less)	120 (32.4)	94 (28.0)
	Medium (Post-secondary, not university)	163 (43.9)	130 (38.7)
	High (Bachelor degree or higher)	88 (23.7)	112 (33.3)
	**Adult socio-economic status at age 26**^a^	**371 (100.0)**	**334 (100.0)**
	Low	103 (27.8)	58 (17.4)
	Medium	194 (52.3)	195 (58.4)
	High	74 (20.0)	81 (24.3)
*From 32–37*	**Total assessed on first birth with no birth by age 31**^a^	**263 (100.0)**	**204 (100.0)**
	**First birth from 32–37**	**121 (46.0)**	**94 (46.1)**
	**Level of education at age 32**^a^	**263 (100.0)**	**204 (100.0)**
	Low (High school or less)	71 (27.0)	48 (23.5)
	Medium (Post-secondary, not university)	115 (43.7)	74 (36.3)
	High (Bachelor degree or higher)	77 (29.3)	82 (40.2)
	**Adult socio-economic status at age 32**^a^	**263 (100.0)**	**204 (100.0)**
	Low	73 (27.8)	47 (23.0)
	Medium	135 (51.3)	118 (57.8)
	High	55 (20.9)	39 (19.1)
*At age 38*	**Total assessed on first birth with no birth by age 37**^a^	**142 (100.0)**	**110 (100.0)**
	**Birth at age 38**	**3 (2.1)**	**1 (0.9)**
	**Want a child in future**^c^	**139 (100.0)**	**109 (100.0)**
	No	31 (22.5)	33 (30.3)
	Yes	73 (52.9)	51 (46.8)
	Unsure	34 (24.6)	25 (22.9)

Columns may not sum to 100.0% due to rounding.

^a^ Total with information for each characteristic

^b^ For level of education at age 21 this was based on the highest high-school qualification obtained.

^c^ Those with a birth at age 38 excluded.

**Fig 3:**
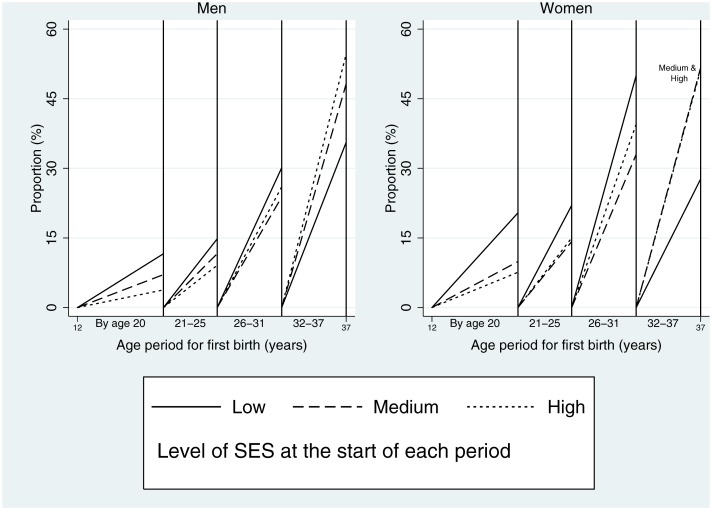
Proportion of men and women with a first live birth in four age periods by socioeconomic status (SES). Denominator in each period is those with no birth in a prior period. For the first age period childhood SES of the family is used.

Fig 3 shows the proportion to report a first birth in four age periods by socioeconomic status at the start of each period, for those who did not report a first birth in a prior age period (hence at the start of each age period the initial proportion returns to zero). For first birth by age 20, there is a gradient with childhood SES, such that a higher proportion of those with lower socioeconomic status reported a first birth. This was true for both men and women, and more pronounced for women. A higher proportion of those with lower socioeconomic status also reported first births from age 21–25, and from 26–31. However, in the final age period, the pattern reversed and a higher proportion of those from the highest socioeconomic status reported a first birth.

**Fig 4:**
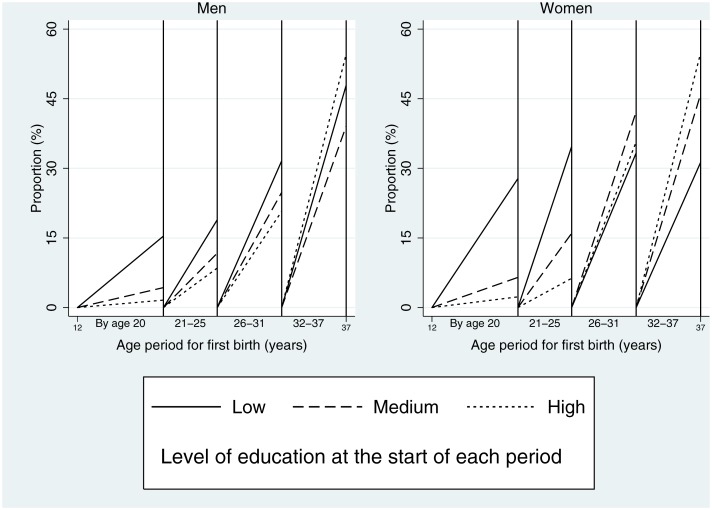
Proportion of men and women with a first live birth in four age periods by level of education. Denominator in each period is those with no birth in a prior period.

Fig 4 considers the relationship with first birth and level of education at the start of each period. A much higher proportion of those with lower levels of education at age 21 reported a first birth by age 20 than for other levels of education, with a gradient present. This association persisted for first birth from age 21–25 for both sexes and from age 26–31 for men. The pattern shifted such that a higher proportion of those with the highest level of education reported a first birth from 32–37, and for women the relative positioning by education had begun to shift from age 26–31.

Table 2 quantifies the relationships in the Figures and confirms that first birth by age 20 in women is strongly predicted by childhood SES, and that the relationship with adult socioeconomic status reverses in the oldest age period. A significantly much lower proportion of women from families of medium (RR = 0.48, 95% CI 0.29–0.81) and high (RR = 0.37, 95% CI 0.16–0.88) socioeconomic status reported a first birth than from families with low socioeconomic status, with a gradient evident. A similar pattern was observed for men, but was not statistically significant: RR = 0.61, 95% CI 0.31–1.2 for medium and RR = 0.33, 95% CI 0.09–1.1 for high socioeconomic status compared with low. The strength of this effect weakened across subsequent age periods though the pattern across age periods was not significantly different for women (p-value = 0.2 for interaction) and was of borderline significance for men (p-value = 0.05 for interaction). For adult SES from 32–37 the strong reversal indicated in the figure was confirmed for women, with a much higher proportion of those from medium (RR = 1.8, 95% CI 1.1–3.0) and high (RR = 1.9, 95% CI 1.1–3.3) socioeconomic status reporting a first birth compared with low. For men, the observed pattern was similar, but not statistically significant (RR = 1.4, 95% CI 0.95–2.0 and RR = 1.5, 95% CI 0.99–2.1 respectively). The pattern across age periods was significantly different for women (p-value = 0.005 for interaction), though not for men (p-value = 0.1 for interaction)

**Table 2:** Relative Risk (RR) of the occurrence of first birth according to socioeconomic factors at the start of each age period.

Characteristic	Age period for first birth^a^			
	To age 21	From 21–25	From 26–31	From 32–37
	RR (95% CI)	RR (95% CI)	RR (95% CI)	RR (95% CI)
*Men*				
**Childhood SES**				
Low	1.0	1.0	1.0	1.0
Medium	0.61 (0.31–1.2)	0.70 (0.40–1.2)	1.2 (0.72–1.9)	1.0 (0.68–1.5)
High	0.33 (0.09–1.1)	0.46 (0.19–1.1)	0.67 (0.33–1.3)	1.3 (0.84–1.9)
**Level of education**^b^				
Low	1.0	1.0	1.0	1.0
Medium	0.28 (0.13–0.60)	0.63 (0.36–1.1)	0.80 (0.54–1.2)	0.89 (0.64–1.2)
High	0.10 (0.02–0.42)	0.45 (0.22–0.90)	0.66 (0.41–1.1)	1.1 (0.80–1.5)
**Adult SES**^b^				
Low		1.0	1.0	1.0
Medium		0.81 (0.49–1.3)	0.77 (0.52–1.1)	1.4 (0.95–2.0)
High		0.64 (0.16–2.5)	0.85 (0.52–1.4)	1.5 (0.99–2.1)
***Women***				
**Childhood SES**				
Low	1.0	1.0	1.0	1.0
Medium	0.48 (0.29–0.81)	0.59 (0.37–0.94)	0.80 (0.58–1.1)	1.1 (0.71–1.8)
High	0.37 (0.16–0.88)	0.61 (0.31–1.2)	0.67 (0.41–1.1)	1.0 (0.58–1.7)
**Level of education**^b^				
Low	1.0	1.0	1.0	1.0
Medium	0.24 (0.13–0.44)	0.47 (0.30–0.72)	1.3 (0.88–1.8)	1.4 (0.87–2.2)
High	0.08 (0.03–0.27)	0.18 (0.09–0.38)	1.1 (0.74–1.6)	1.7 (1.1–2.6)
**Adult SES**^b^				
Low		1.0	1.0	1.0
Medium		0.66 (0.43–1.0)	0.68 (0.49–0.95)	1.8 (1.1–3.0)
High		0.67 (0.23–2.0)	0.82 (0.56–1.2)	1.9 (1.1–3.3)

P-value for interaction across age period for men: childhood SES 0.05; level of education 0.002; adult SES 0.1; for women: childhood SES 0.2, level of education <0.0001, adult SES 0.005.

^a^ Denominator in each age period is those who have not had a first birth in a prior age period.

^b^ Characteristic at the start of each age period

Table 2 also indicates a much lower proportion of men and women with higher levels of education reported a first birth by age 20, and from 21–25. For first birth by age 20, a much lower proportion of men with a medium (RR = 0.28, 95% CI 0.13–0.60) and high (RR = 0.10, 95% CI 0.02–0.42) level of education reported a first birth compared with a low level. For women, the respective rates were RR = 0.24 (95% CI 0.13–0.44) and RR = 0.37 (95%CI 0.16–0.88). Relative Risks remained lowered for both sexes from 21–26, then weakened for men for first birth from 26–31 and 32–37. For women, there was no evidence of a difference by level of education for first birth from 26–31; however, for first birth from 32–37 the pattern reversed and a higher proportion of women with medium (RR = 1.4, 95% CI 0.87–2.2) and high (RR = 1.7, 95% CI 1.1–2.6) levels of education reported a first birth compared with low. The pattern by age period differed significantly for both women (p-value<0.0001 for interaction) and for men (p-value = 0.002 for interaction).

Table 3 assesses the independent effects of childhood SES and adult SES on first birth (Model 1), and then adult SES and level of education (Model 2), using multivariable models that included both factors. Low childhood SES was a strong driver of first birth by age 20, but did not continue to impact first birth in later age periods (Model 1). In contrast, higher adult SES remained strongly associated with higher first birth rates from age 32–37, after adjustment for childhood SES. Including both adult SES and level of education did not substantially alter the findings, indicating that overall these relationships were operating largely independently of one another (Model 2). The exception was that for women from 32–37, the increased relative risk for first birth with higher levels of education was weakened and no longer significantly higher when adult SES was included as a covariate.

**Table 3:** Multivariate models of the occurrence of first birth within each age period to assess independent effects of a) childhood SES and adult SES at the start of each period (Model 1); and b) adult SES and level of education at the start of each period (Model 2).

Characteristic	Age period for first birth^a^		
	From 21–25	From 26–31	From 32–37
	RR (95% CI)	RR (95% CI)	RR (95% CI)
***Men***			
***Model 1***			
**Childhood SES**			
Low	1.0	1.0	1.0
Medium	0.68 (0.38–1.2)	1.2 (0.75–1.9)	0.94 (0.64–1.4)
High	0.46 (0.18–1.2)	0.69 (0.35–1.4)	1.2 (0.77–1.7)
**Adult SES**^b^			
Low	1.0	1.0	1.0
Medium	0.85 (0.50–1.4)	0.76 (0.52–1.1)	1.4 (0.97–1.9)
High	0.78 (0.19–3.1)	0.90 (0.55–1.5)	1.4 (0.96–2.1)
***Model 2***			
**Adult SES**^b^			
Low	1.0	1.0	1.0
Medium	0.85 (0.51–1.4)	0.81 (0.55–1.2)	1.5 (1.0–2.1)
High	0.90 (0.22–3.6)	1.2 (0.67–2.0)	1.4 (0.94–2.2)
**Level of education**^b^			
Low	1.0	1.0	1.0
Medium	0.65 (0.38–1.1)	0.81 (0.55–1.2)	0.81 (0.58–1.1)
High	0.42 (0.19–0.90)	0.58 (0.33–1.0)	0.94 (0.67–1.3)
***Women***			
***Model 1***			
**Childhood SES**			
Low	1.0	1.0	1.0
Medium	0.60 (0.37–0.98)	0.79 (0.57–1.1)	1.0 (0.66–1.6)
High	0.62 (0.32–1.2)	0.67 (0.41–1.1)	0.88 (0.52–1.5)
**Adult SES**^b^			
Low	1.0	1.0	1.0
Medium	0.69 (0.44–1.1)	0.72 (0.51–1.0)	1.9 (1.2–3.1)
High	0.81 (0.27–2.4)	0.91 (0.61–1.3)	2.0 (1.2–3.5)
***Model 2***			
**Adult SES**^b^			
Low	1.0	1.0	1.0
Medium	0.63 (0.41–0.97)	0.68 (0.49–0.95)	1.7 (1.0–2.8)
High	0.90 (0.33–2.5)	0.84 (0.55–1.3)	1.7 (0.95–3.0)
**Level of education**^b^			
Low	1.0	1.0	1.0
Medium	0.47 (0.30–0.72)	1.2 (0.87–1.8)	1.2 (0.73–1.9)
High	0.18 (0.08–0.37)	1.1 (0.69–1.6)	1.4 (0.84–2.2)

^a^ Denominator in each age period is those who have not had a first birth in a prior age period.

^b^ Characteristic at the start of each age period

## Discussion

In this birth cohort, only 69% of men and 76% of women had become parents by age 37, and there was substantial variation in the timing of first births influenced by socioeconomic characteristics across the life-course. Lower socioeconomic status in childhood was strongly associated with adolescent parenthood. While this early effect continued to influence the cumulative incidence of who became a parent to age 31 for men, and to age 37 for women, socioeconomic status in childhood did not have a large influence on entry into parenthood after age 21. Lower adult socioeconomic status was associated with entry into parenthood in younger age periods, but this pattern *reversed* at older ages. By the mid to late thirties, higher adult socioeconomic status was associated with a greater likelihood of first becoming a parent. Higher levels of education strongly delayed first birth in the early twenties, but again the pattern reversed at older ages. Thus socioeconomic circumstances at each age appeared to act most strongly on first births at that age, but with different effects at different ages. These patterns with socioeconomic circumstances were stronger for women than for men.

Particular strengths of the study include the longitudinal design using a birth cohort with data for both sexes the representativeness of the sample, and the extremely high retention at each assessment. Socioeconomic data were collected prospectively across the life-course, including information on the family of origin collected at several assessments in childhood limiting recall bias. Such data were restricted to events that occurred prior to each interval for first birth, with one exception. As our measure of education for first birth prior to 21 was highest level of qualification at age 21, the much higher likelihood of first birth for those with low levels of education can not be interpreted as causal, and likely reflects in part lower educational level due to childbearing. Otherwise the collection of data on socioeconomic factors and education prior to births enables the causal direction to be established. In this cohort, 24% of women had not a birth by age 37, slightly higher than recent estimates that 19–21% of women aged 35–39 in Australia, Canada, New Zealand, the United Kingdom and the United States remain childless [46].

Limitations include the reliance on self-reports at 5 to 6 year intervals, as faulty recall of timing of reproductive events could result in misclassification, as could limitations of men’s knowledge of their partners’ reproductive histories. However, the collection of information on reproductive events at multiple assessments enabled better estimates than those obtained from one period alone. Larger studies are required to confirm findings for men. The scales used to ascertain socioeconomic status changed from the Elley-Irving scale to the NZSEI, which could have resulted in some individuals changing categories between assessments; however both are similarly constructed occupational scales and this could only account for a portion of the differences between measures at age 26 and 32, and not the considerable movement between the other ages.

The substantial movement in individual socioeconomic status over the life-course, as well as from the status of their family in childhood, confirms the importance of using multiple measures preceding first births–as in this study—when considering the impact on entry into parenthood, particularly as having children can result in changes to socioeconomic status. This highlights the limitation of cross sectional studies that measure socioeconomic characteristics only at the time of interview, and thus may give a distorted picture of these relationships.

This study is able to show a long term effect of childhood socioeconomic status on earlier entry into parenthood to age 31 for men and 37 for women. However, it also demonstrates that the observed association at older ages was largely driven by the strong relationship between lower childhood socioeconomic status and who had become a parent by age 21. Past that age, using age specific birth rates in three different age periods, both with and without adjusting for adult socioeconomic status, the effect was weaker and not statistically significant from age 21 to age 31, and was not evident past age 31. Thus, while there may be a role for a shared norm of earlier childbearing for those from families of lower socioeconomic status, perhaps through intergenerational transmission of age at first birth; that this effect does not continue suggests that differences in age of sexual initiation, sexual partnering and contraceptive use may be more important in understanding this early difference (van Roode 2012) [39].

Low adult socioeconomic status was, as expected, also associated with parenthood before age 26, though our findings were not statistically significant. However, we demonstrated a strong reversal in the direction of the effect for socioeconomic status, with birth rates in the mid to late thirties for women from higher socioeconomic status double those from lower socioeconomic status. The relationship was less marked for men, and we speculate that this reversal may be more evident in the next age period after age 37, because the normative age for parenthood is older for men. Overall the findings are consistent with a later normative age for parenthood, with delayed parenthood past age 31, and an accompanying recuperation period in the mid to late thirties. Furthermore, our results demonstrate that this effect of adult socioeconomic status operated largely independently of childhood socioeconomic status.

Higher educational level was confirmed as a strong deterrent to entry into parenthood for both sexes in the early twenties. As for socioeconomic status, this relationship with education was reversed in the mid to late thirties such that women with higher levels of education had higher birth rates at this age, supporting a later norm for family formation, rather than a forgoing of children altogether. Again, that this relationship was not supported for men, may be because the normative age for men with higher education to enter into parenthood is older than examined here. Consideration of the relative impact of socioeconomic status and education indicates that education is more important in understanding the deferment that occurs in the early twenties, whereas socioeconomic status was more important in driving parenthood in the mid to late thirties. As our early measure of level of education indicated who had a university bursary, the deferment in the early twenties associated with education would support that enrolment in education is not considered compatible with having children [1].

This longitudinal analysis demonstrates a strong reproductive polarisation in the timing of parenthood, with those experiencing greater disadvantage in society having children at a younger age and those who are more advantaged waiting until they are older. This differential in a cohort born in New Zealand, a country characterised by comparatively limited social policies to assist families, would support the association between such liberal policies and increased heterogeneity in first birth [10,47]. This polarisation is likely to result in greater disadvantage for the next generation of children as the commonality of motherhood becomes age segregated by socioeconomic circumstances. Indeed, previous research suggests there is already widening disparity for children, with those born to young mothers now with greater socioeconomic disadvantage compared to prior generations [5,48]. Thus greater assistance for young disadvantaged families is required to mitigate the effect of this current disparity and to unlink childhood disadvantage from early childbearing.

In contrast, the tendency to delay parenthood into the mid to late thirties for those with higher levels of education and with higher socioeconomic status indicates that current social norms and policies are encouraging many people to delay parenthood to an age where natural fertility is declining. While there is some recuperation in the mid to late thirties, our findings indicate that many men and women still wished to have a child or more children in the future. As fertility declines with age, and fertility treatments are less successful at older ages, this may result in a substantial proportion who will not be able to become parents or not have as many children as they desire [21]. Government policies that facilitate parenthood during more optimal reproductive years may be helpful in addressing these issues [49,50].

Future research on this cohort has been planned to investigate how socioeconomic characteristics influence total number of children at the end of the reproductive span, and the proportions who remain voluntarily and involuntarily childless. Proximate factors associated with timing of parenthood, especially patterns of sexual partnering, have already been examined in this cohort [39]. They are likely to mediate some of the relationships observed. Linking SES measures to such proximate factors is beyond the scope of this paper but could be undertaken to investigate specific hypotheses in the future. Furthermore, other characteristics of the family of origin such as the age of the individual’s mother at her first birth could further inform about what drives these observed fertility patterns.
